# An iterative deconvolution model to extract the temporal firing properties of the auditory nerve fibers in human eCAPs

**DOI:** 10.1016/j.mex.2021.101240

**Published:** 2021-01-22

**Authors:** Yu Dong, H. Christiaan Stronks, Jeroen J. Briaire, Johan H.M. Frijns

**Affiliations:** a ENT-Department, Leiden University Medical Centre, PO Box 9600, 2300, RC Leiden, the Netherlands; b Leiden Institute for Brain and Cognition, PO Box 9600, 2300, RC Leiden, the Netherlands

**Keywords:** Cochlear implants, Sensorineural hearing loss, Electrically evoked compound action potential, Deconvolution, Unitary response, Temporal properties

## Abstract

The electrically evoked compound action potential (eCAP) has been widely studied for its clinical value for the evaluation of the surviving auditory nerve (AN) cells. However, many unknowns remain about the temporal firing properties of the AN fibers that underlie the eCAP in CI recipients. These temporal properties may contain valuable information about the condition of the AN. Here, we propose an iterative deconvolution model for estimating the human evoked unitary response (UR) and for extracting the compound discharge latency distribution (CDLD) from eCAP recordings, under the assumption that all AN fibers have the same UR. In this model, an eCAP is modeled by convolving a parameterized UR and a parameterized CDLD model. Both the UR and CDLD are optimized with an iterative deconvolution fitting error minimization routine to minimize the error between the modeled eCAP and the recorded eCAP.

• This method first estimates the human UR from eCAP recordings. The human eCAP is unknown at the time of this writing. The UR is subsequently used to extract the underlying temporal neural excitation pattern (the CDLD) that reflects the contributions from individual AN fibers in human eCAPs.
• By calculating the CDLD, the synchronicity of AN fibers can be evaluated.

## Graphical abstract

**Figure:**
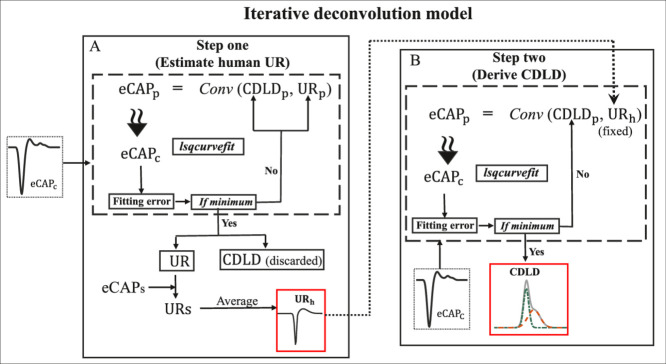

Specifications table

**Table:** 

Subject Area:	Neuroscience
More specific subject area:	Cochlear implants
Method name:	An iterative deconvolution model for extracting temporal firing properties of the auditory nerve in human eCAPs
Name and reference of original method:	Y. Dong, J. J. Briaire, J. D. Biesheuvel, H. C. Stronks, and J. H. M. Frijns, Unravelling the Temporal Properties of Human eCAPs through an Iterative Deconvolution Model, Hear. Res., vol. 395, p. 108037, 2020, doi:10.1016/j.heares.2020.108037. https://doi.org/10.1016/j.heares.2020.108037
Resource availability	

**Method details**

## Background

A cochlear implant (CI) is an intracochlear device that can restore hearing with direct electrical stimulation of the auditory nerve (AN). A CI can also be applied to measure electrically evoked AN responses using the reverse telemetry function. Typically, AN activity is evoked with short electrical pulses, and the response comprises the superposition of many action potentials from AN fibers over time. This response is called the electrically evoked compound action potential (eCAP). To date, single-fiber action potentials have not been recorded from the human AN. ECAP recordings can provide information on the amplitude and latency of the evoked compound AN response, but they do not provide information about the underlying excitation patterns of individual AN fibers. Clinically, the eCAP is typically evaluated by examining the main peaks of the eCAP; i.e., the first negative peak (N1) and the first positive peak (P1) [12,13]. Previously, animal studies have reported that the eCAP waveform was dependent on both the number of action potentials and the degree of synchronicity in the AN fiber population [6,^12^,16]. The temporal firing properties in eCAPs can potentially reflect additional, valuable information, such as the survival of AN fibers [7,12]. However, extracting the temporal firing properties of single fibers directly from the eCAP is mathematically complex. As a result, these properties are often overlooked [1], [2], [3], [4]. Here we propose a method to extract the temporal firing properties of the AN fibers in eCAPs. The procedure is based on the findings of our previous study [14].

**Fig. 1:**
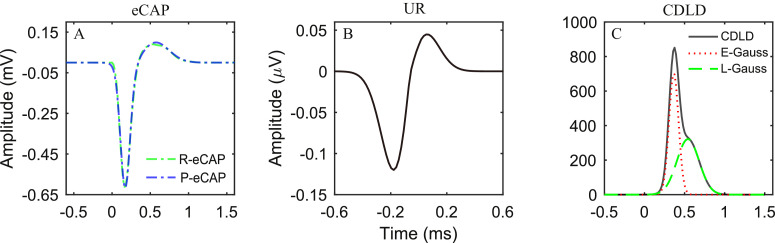
An example of the deconvolution model. (A) According to Eq. (1), the recorded electrically evoked compound action potential (R-eCAP, green interrupted line) was predicted (P-eCAP, blue interrupted line) with the convolution of (B) a UR model (also see Eq. (3)) and (C) a compound discharge latency distribution (CDLD) model (blue line, see Eq. (4)), by implementing the deconvolution fitting error minimization routine. The CDLD model consists of two Gaussian components: the early Gaussian component (E-Gauss, red dotted line) and the late Gaussian component (L-Gauss, green dashed line).

The action potential generated by a single fiber can be registered by a recording electrode and is called the unitary response (UR). The UR is generally thought to be constant, and all URs are assumed to contribute equally to the acoustically evoked CAP [5,6]. We assume this concept also holds for eCAPs [7,^8^,14] because the eCAP also represents a superposition of a series of action potentials from individual AN fibers in response to an electric stimulus over time. Thus, based on these assumptions, we describe the eCAP as the convolution of many URs with a compound discharge latency distribution (CDLD), according to Eq. (1) (see also Fig. 1):

(1) $$\text{eCAP}\left(t\right)=\int_{-\infty }^{t}\text{CDLD}\left(\tau \right)*\text{UR}\left(t-\tau \right)d\tau$$

Here, the CDLD is the probability density function, *t* is time, and τ is the variable of integration. The CDLD weighs all URs of each excited AN fiber across time and reflects the neural synchronicity (i.e., the temporal properties). Thus, the temporal firing properties of the AN fibers in eCAPs can be captured from the CDLDs. Mathematically, the CDLD cannot assume negative values, and the area under a CDLD curve reflects the number of excited AN fibers.

The only study on the human CDLD was conducted by Strahl et al. [7]. They predicted the CDLD by a direct deconvolution of the human eCAP using the guinea pig UR (UR_gp_) [7]. The deconvolution was performed with Eq. (2):

(2) $$\text{CDLD}\left(t\right)=\;F^{-1}\left[\frac{F\left(\text{eCAP}\left(t\right)\right)}{F\left(\text{UR}\left(t\right)\right)}\right]$$

Here, *t* is time, *F* represents the Fourier transform, and $F^{-1}$ represents the inverse Fourier transform. Strahl et al. observed a CDLD with two Gaussian components, which could be attributed to two separate groups of neural responses. However, when we reproduced their method on human patient data (see details in [14]) we obtained unrealistic CDLDs because they contained negative phases and high-frequency components. To suppress these negative phases and high-frequency components, Strahl et al. filtered the CDLD with a 2.5 kHz low-pass filter and shifted the CDLD upward. However, this post-processing might have compromised the validity of the CDLD in its ability to reflect the temporal firing properties of the AN.

To facilitate a direct deconvolution of the human eCAP into a CDLD to describe the temporal firing properties of AN fibers underlying the eCAP, Strahl et al. used the UR_gp_, because the human UR (UR_h_) was, and still is unknown. However, there are several anatomical differences between the two species that potentially can affect the shape of the UR. There are differences in the size and shape of the cochlea [11,14] and the spiral ganglion cell body is myelinated in guinea pigs, but not in humans [17]. Moreover, eCAP recordings in humans are usually performed at intracochlear sites, e.g., [7,^8^,14], whereas the UR_gp_ used in [7] was recorded at the round window niche [6]. The application of a direct deconvolution of the human eCAP into a CDLD using the UR_gp_ can thus be expected to yield a less valid CDLD.

**Fig. 2:**
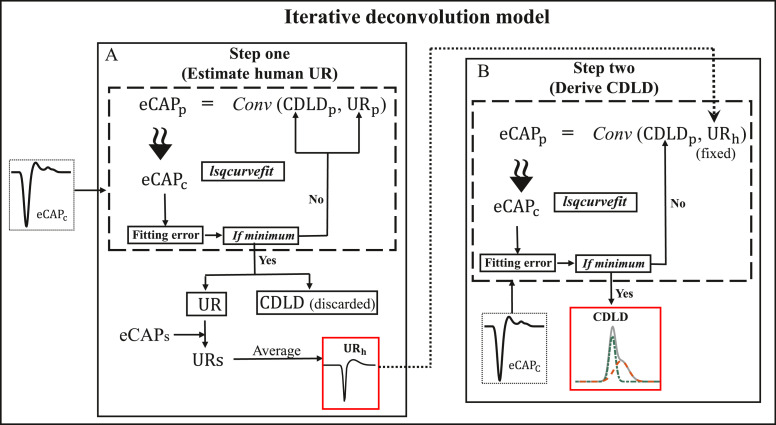
Iterative deconvolution model workflow. The recorded electrically evoked action potential (eCAP) waveforms are pre-processed and used as the input for the deconvolution fitting error minimization routine (DMR, enclosed in the dashed square) in both step one and step two. This DMR is conducted by the lsqcurvefit function provided in MATLAB. In the DMR, the predicted eCAP ($\text{eCA}P_{P}$) is calculated by the convolution of the parameterized unitary response (UR) model ($UR_{p}$) with the parameterized compound discharge latency distribution (CDLD) model ($\text{CDL}D_{p}$). (A) In step one, both the $UR_{p}$ and the $\text{CDL}D_{p}$ are optimized with the DMR to achieve an approximate match between the $\text{eCA}P_{P}$ and the baseline-corrected ($\text{eCA}P_{C}$). When the fitting error (i.e., the difference between the $\text{eCA}P_{P}$ and the $\text{eCA}P_{C}$) reaches the minimum, the UR and CDLD are obtained. In this step, each eCAP generates a UR. The URs are obtained from a series of eCAPs, and the average of these URs is defined as the human UR ($UR_{h}$, enclosed in the red square). (B) In step two, the $UR_{h}$ is fixed, and only the $\text{CDL}D_{p}$ is iteratively adjusted with the DMR to generate the best fitting CDLD for each individual eCAP (CDLD, enclosed in the red square). *Conv* represents the convolution function in MATLAB.

To overcome these problems we propose an iterative deconvolution model to simulate the deconvolution computation. The recorded eCAPs are entered as input for this model to obtain the UR_h_ and the corresponding CDLDs. This model consists of a two-step procedure (Fig. 2). It estimates the UR_h_ in step one (Fig. 2A) and derives the temporal firing properties of AN fibers underlying the eCAP in step two (Fig. 2B), without the need for any post-processing of the CDLD. In both steps, an eCAP is modeled by convolving a UR model with a CDLD model. Then, the modeled eCAP is optimized by iteratively adjusting the variables in the parameterized UR and CDLD models, until the modeled eCAP matches the recorded eCAPs. In step one, the descriptive parameters of both the UR and CDLD model are variable. After optimization, an estimate of the UR_h_ and CDLD is obtained for each eCAP waveform available. A unified UR_h_ is subsequently estimated by averaging the available collection of individual UR_h_s (Fig. 2A). Using the unified UR_h_ obtained in step one, a similar procedure is used in step two where only the CDLD parameters are iteratively varied (Fig. 2B). The resulting CDLDs can reveal the temporal firing properties of AN. More detailed information about the deconvolution model is given in the below sections.

## Model construction of the UR and CDLD

According to Eq. (1), the UR and the CDLD are required to simulate the recorded eCAPs. At present, the UR_h_ has not been described with electrophysiological recordings. Because the UR_h_ might be different from the UR_gp_ [11] or other animals, it is preferable to estimate the UR_h_ [14]. As a starting point, we used the UR_gp_ function reported in [6] to estimate the UR_h_. For this purpose, the UR was parameterized as shown in Eq. (3).

(3) $$UR_{p}\left(t\right)=\frac{U}{\sigma }\left(t-t_{0}\right)e^{\left[-\frac{{\left(t-t_{0}\right)}^{2}}{2\sigma^{2}}\right]}$$

The UR model consists of a negative (N) and positive (P) phase; the transition point between the negative component and the positive component is defined as $t_{0}$. Hence, for *t* < $t_{0}$, the magnitude, *U* (V), of the negative peak is $U_{N}$, and the width, *σ* (sec), of the negative component is $\sigma_{N}$; and for *t* > $t_{0}$, the magnitude of the positive peak is $U_{P}$ and the width of the positive component is $\sigma_{P}$. Boundary limits for the variables in Eq. (3) are introduced to constrain the solutions (see details in step one, Fig. 2).

Earlier studies have observed eCAPs with two positive peaks, which might originate from two separate groups of neural responses [12,13]. Consistent with Strahl et al. [7], our method implements a parameterization of the CDLD with a mixture of two Gaussian components, as shown in Eq. (4) (also see Fig. 1C).

(4) $$\text{CDL}D_{p}=\alpha_{1}*N\left(\mu_{1},\;\sigma_{1}\right)+\alpha_{2}*N\left(\mu_{2},\;\sigma_{2}\right)$$

where *N* is a Gaussian distribution; the variables $\alpha_{1},\;\mu_{1}$ and $\sigma_{1}$ belong to the early Gaussian component (in time), and the variables, $\alpha_{2},\;\mu_{2}$ and $\sigma_{2}$ belong to the late Gaussian component. The variables $\alpha_{1}$ and $\alpha_{2}$ represent the peak amplitudes; $\mu_{1}$ and $\mu_{2}$ are the peak latencies; and $\sigma_{1}$ and $\sigma_{2}$ represent the peak widths. Similar to Eq. (3), boundary limits for the variables in Eq. (4) are set to constrain the solutions (see details in step one and two, Fig. 2). Details are given below.

### Optimization routine

The procedure described here involves the application of a deconvolution fitting error minimization routine (DMR) in step one and two. The parameterized UR model (UR_p_, Eq. (3)) and the parameterized CDLD model (CDLD_p_, Eq. (4)) are used to predict a recorded eCAP waveform using indirect deconvolution. This indirect procedure uses a convolution step to estimate UR_h_ and CDLD by implementing DMR to optimize the match between eCAP_p_ and the recorded eCAP. The initial values and boundary limits of the UR_p_ and CDLD_p_ parameters must be assigned for the DMR to run. Then, the eCAP_p_ and the baseline-corrected eCAP (eCAP_c_) (see details in the next section), initial values and boundary limits of the UR_p_ and CDLD_p_ parameters are used as input into the DMR. The DMR iteratively manipulates the parameters of the UR_p_ and the CDLD_p_ in step one (Fig. 2A), or only the CDLD_p_ in step two (Fig. 2B) within the boundary limits to minimize the fitting error (see Fig. 2) with the lsqcurvefit function provided in MATLAB (Mathworks 2016a, Natick, MA, USA). The fitting error refers to the difference between the eCAP_p_ and the eCAP_c._ Accordingly, the eCAP_p_ gradually converges to the eCAP_c_. When the fitting error reaches the minimum value (i.e., the eCAP_p_ optimally approximates the eCAP_c_), the DMR outputs the values of the UR_p_ and CDLD_p_ parameters. With these values, the UR and CDLD (step one) and subsequently the final CDLD estimate (step two) can be generated. The MATLAB script of this DMR is attached in the supplementary material of this publication.

### Extraction of temporal AN firing properties from eCAPs

In this section, we will describe the workflow to calculate the CDLD from recorded eCAP waveforms. Before any analysis can be performed, the raw eCAP waveforms have to be pre-processed. First, a baseline correction is carried out. The eCAP tail can be used to determine the baseline, because neural responses and any remaining artifacts are not expected to be present in this part of the eCAP waveform. At approximately 1.5 ms after stimulus artifact a reliable baseline estimate can be obtained [9,14]. The baseline correction is performed by subtracting the average amplitude of the tail section from the eCAP waveform. In addition, we have observed that performing a convolution on a finite-length signal typically introduces distortions at the leading and trailing ends of the signal. To prevent distortion of the eCAP waveforms, signal extensions can be deployed [15] by adding 50 samples to the start and end of each waveform. This is realized by performing a linear extrapolation to baseline. This extrapolation only affects the CDLD before and after the recording window [6,^14^,15].

The two steps proposed for deriving the temporal firing properties of the AN from eCAPs are shown in Fig. 2. Before the CDLD can be determined, the UR_h_ has to be estimated from the available eCAP dataset with the DMR (Fig. 2A). In step one (Fig. 2A), the parameters of both the UR_p_ (Eq. (3)) and the CDLD_p_ (Eq. (4)) are variable and will be optimized by the DMR. This ensures that the eCAP_p_ optimally matches the baseline-corrected eCAP (eCAP_c_). After the last iteration, the UR and CDLD of the optimal eCAP_p_ are derived. In our data set [14] a series of eCAPs were recorded at different electrode contacts with different stimulus levels from different subjects. According to the assumption that the UR is identical in all contributing AN fibers and across electrode contacts, stimulus levels and subjects [7,^8^,14], a representative human UR can be estimated by averaging all these URs obtained from a series of eCAPs. The UR model and the CDLD model can interact freely in step one; thus, the temporal firing properties can be manifested in both the UR and the CDLD. Consequently, the resulting CDLDs do not accurately reflect the temporal information in eCAPs and these CDLDs are discarded and re-calculated by using a constant UR, as outlined below.

As mentioned in the Model Construction section, the initial values and boundary limits of the parameters have to be assigned before performing the DMR. Because the UR_h_ and UR_gp_ are expected to be similar [10], we used the morphological parameters of the UR_gp_ as a reference for the UR_h_ [6]. Accordingly, the UR and CDLD outcomes were constrained with the following domain values [14]: $U_{N}$ [0.02, 0.25], $\sigma_{N}$ [0.02, 0.13], $U_{P}$ [0, 0.12], $\sigma_{P}$ [0.08, 0.25], $t_{0}$ [−0.25, 0.06],$\;\alpha_{1}$ [0, 0.35], $\mu_{1}$ [0.04, 1.3], $\sigma_{1}$ [0, 0.3], $\alpha_{2}$ [0, 0.35], $\mu_{2}$ [0.04, 1.3], $\sigma_{2}$ [0, 0.3]. Based on the parameters of UR_gp_, the initial starting values of the DMR parameters are set to: $U_{N}$ (0.12), $\sigma_{N}$ (0.045), $U_{P}$ (0.06), $\sigma_{P}$ (0.12), $t_{0}$ (−0.06),$\;\alpha_{1}$ (0.08), $\mu_{1}$ (0.38), $\sigma_{1}$ (0.06), $\alpha_{2}$ (0.05), $\mu_{2}$ (0.5), $\sigma_{2}$ (0.14). Then, the parameters of the UR model (Eq. (3)) and the CDLD model (Eq. (4)) are iteratively manipulated simultaneously with the DMR, until they approximate the recorded eCAPs (Fig. 1, green line).

Setting appropriate starting values and boundaries for the DMR parameters is necessary, both to obtain a realistic UR_h_ and CDLD with the DMR and to converge to an optimal eCAP_p_. An important factor to consider when setting the starting values and boundaries for the DMR parameters is the morphology of eCAP recordings, particularly the eCAP waveforms that have the maximal and minimal amplitudes in one's dataset. The morphological characteristics of eCAPs include, but are not limited to the main peak (i.e., the N1 and P1) and, maybe, a second peak (i.e., the N2 and P2) and the corresponding peak latencies, [12,14]. These parameters are influenced by extrinsic factors, including the stimulation level, intra-cochlear test electrode location, the separation between the stimulating and recording electrodes, stimulus polarity, artifact reduction methods, and implant designs [3,12]. For instance, a larger eCAP main peak would most likely require wider boundaries for $\alpha_{1}$ and $\alpha_{2}$, and longer peak latencies would require wider boundaries for $\mu_{1}$ and $\mu_{2}$. Moreover, because the parameter estimates are sensitive to the initial values of the DMR parameters, they should be optimized manually, when needed, to achieve an adequate fit. The goodness of fit to overall data was evaluated by calculating the normalized root mean square error (NRMSE). Therefore, the initial values and boundaries might need to be optimized with different datasets. In [14], the parameters of human UR were estimated: $U_{N}$ = 0.155 µV, $\sigma_{N}$ = 0.038 ms, $U_{P}$ = 0.022 µV, $\sigma_{P}$ = 0.155 ms, $t_{0}$ = −0.128. For the human dataset, this UR can be used directly for step two. Nevertheless, we strongly recommend that researchers should examine the consistency of human UR when using their own datasets.

In step two (Fig. 2B), the temporal firing properties of the AN in human eCAPs are extracted by calculating CDLDs (Fig. 2B). Due to the interaction between the UR_h_ and CDLD (see above), the CDLD calculation with the DMR must use a constant UR_h_. With the fixed UR obtained in step one, the DMR can only adjust the parameters of the CDLD model (Eq. (3)), with the recorded eCAPs as input. Consequently, because the fixed UR_h_ and CDLD model can no longer interact, all the temporal firing properties in eCAPs are driven into CDLDs. Thus, these CDLDs validly reflect the temporal firing properties in the eCAPs. Similar to step one, we constrain the domains for the variables in the CDLD model with the following values: $\alpha_{1}$ [0, 0.35], $\mu_{1}$ [0.15, 1.35], $\sigma_{1}$ [0, 0.45], $\alpha_{2}\;$[0, 0.35], $\mu_{2}$ [0.15, 1.35], $\sigma_{2}$ [0, 0.45]. The starting values of the DMR parameters were set as follows: $\alpha_{1}$ (0.08), $\mu_{1}$ (0.59), $\sigma_{1}$ (0.06), $\alpha_{2}$ (0.05), $\mu_{2}$ (0.6), $\sigma_{2}$ (0.14). The combined boundary limits of these variables allow the model to produce CDLDs without negative phases; thus, unrealistic CDLDs can be avoided without any post-processing. Similar to step one, the starting values and the boundary limits for the CDLD parameters in Eq. (4) can be optimized manually, when needed, to achieve an adequate fit according to the morphology of eCAPs in different datasets.

### Method validation

The validation of the method was discussed in detail in our previous study [14]. In that study, the model presented here was applied to a relatively large data set of human eCAP growth function recordings. This data set consisted of 4982 eCAPs from 111 CI recipients who received a HiRes90K device (Advanced Bionics, Valencia, CA), either with a 1 J or a Mid-Scala electrode array. The eCAPs were recorded measured on eight odd electrode contacts with stimulus levels from 50 to 500 current unit. We have validated both steps of the method.

First, we validated step one, namely the estimation of the UR_h_, by comparing the resulting eCAP_p_s obtained with our estimated UR_h_ [14] to the eCAP_p_s obtained with UR_gp_ [6] in step two. Based on the goodness of fit measure (NRMSE, the normalized root mean square error provided in MATLAB), the eCAPs achieved with UR_h_ were better than those achieved with UR_gp_ [14]. The UR_h_ reduced the fitting error for all eCAPs by approximately 18%. This result supported our assumption that the UR of human AN fibers differs from the UR_gp_ [6]. The assumption that the UR is constant may be contested, as it can hypothetically vary across subjects, electrodes and/or current levels. However, the assumption of UR constancy is necessary, because a fixed UR is needed to optimize the derivation of CDLD in step two. As such, the UR is used solely as a necessary intermediate step to extract a valid CDLD from the eCAP. While a fixed UR is necessary and sufficient for our goal, our deconvolution model can nonetheless be used to investigate whether the UR differs across subjects or different stimulus conditions by running the deconvolution model for each condition separately. However, to more conclusively resolve such questions, direct recordings of the UR_h_ are necessary.

Second, we validated the extraction of the CDLD with the fixed UR by evaluating the goodness of fit of the predicted eCAPs. In general, 93.6% of the recorded eCAPs were predicted accurately, with *a* >0.9 goodness of fit (NRMSE). Thus, these CDLDs provided a good picture of the temporal firing properties of the AN fibers in eCAPs. Importantly, realistic CDLDs were obtained that lacked any negative phases without any post-processing. The remaining 322 eCAPs had deviant waveforms, with relatively small N1 peaks and large P1 peaks; thus, they could not be predicted well with our model (NRMSE <0.9). This may have been caused by the use of a fixed UR that was based on the group-average. This unified UR consisted of a large negative phase and a small positive phase, with a strictly positive CDLD. A UR with this shape could not be used to model the deviant eCAP waveforms with the DMR method (for details, see Dong et al. [14]). However, those cases were fairly rare (6.4%).

Third, we validated the assumption that the CDLD model with two Gaussian components was the optimal model. We designed alternative CDLD models with 1–6 Gaussian components and simulated the recorded eCAPs with the DMR. When the number of Gaussian components increased from 1 to 2, the fitting error diminished substantially (by 78%). When the number of Gaussian components rose from 2 to 6, the fitting outcome remained fairly similar and showed little benefit (error reduced by 7.6%; see Figure 7 in Dong et al. [14]). This result was consistent with the finding of Strahl et al. [7], who also observed CDLDs with two Gaussian components. Taken together, these validations demonstrated that our method can validly unravel the temporal firing properties of the human AN fibers in eCAPs.

## Conclusion

This study proposes an indirect iterative deconvolution model that provides an estimation of the human UR and derives the underlying neural excitation pattern that reflects the contributions from individual AN fibers to human eCAPs. The observed CDLD with two Gaussian components can be attributed to two separate neural response components, which cannot be easily identified in the raw eCAP waveforms.

## Declaration of Competing Interest

The Authors confirm that there are no conflicts of interest.

## Appendix Supplementary materials

Image, application 1

## References

[1] Khan A.M., Whiten D.M., Nadol J.B., Eddington D.K. (2005). Histopathology of human cochlear implants: correlation of psychophysical and anatomical measures. Hear. Res..

[2] Fayad J.N., Linthicum F.H. (2006). Multichannel cochlear implants: relation of histopathology to performance. Laryngoscope.

[3] Ramekers D., Versnel H., Strahl S.B., Smeets E.M., Klis S.F.L., Grolman W. (2014). Auditory-nerve responses to varied inter-phase gap and phase duration of the electric pulse stimulus as predictors for neuronal degeneration. JARO - J. Assoc. Res. Otolaryngol..

[4] Seyyedi Mohammad, Viana Lucas M., Nadol J.B. (2016). Within-subject comparison of word recognition and spiral ganglion cell count in bilateral cochlear implant recipients Mohammad. Physiol. Behav..

[5] Goldstein N.Y.S., M. H., Kiang (1958). Synchrony of neural activity in electric responses evoked by transient acoustic stimuli. Jasa.

[6] Versnel H., Prijs V.F., Schoonhoven R. (1992). Round-window recorded potential of single-fibre discharge (unit response) in normal and noise-damaged cochleas. Hear. Res..

[7] Strahl S.B., Ramekers D., Marjolijn M. B. Nagelkerke K.E.S., Spitzer P., Klis S.F.L., Grolman W., Versnel H. (2016). Assessing the firing properties of the electrically stimulated auditory nerve using a convolution model. Adv Exp Med Biol.

[8] van Gendt M.J., Briaire J.J., Frijns J.H.M. (2019). Effect of neural adaptation and degeneration on pulse-train ECAPs: a model study. Hear. Res..

[9] de Sauvage R.C., Aran J.M., Erre J.P. (1987). Mathematical analysis of VIIIth nerve cap with a linearly-fitted experimental unit response. Hear. Res..

[10] Briaire J.J., Frijns J.H.M. (2005). Unraveling the electrically evoked compound action potential. Hear. Res..

[11] Nadol J.B. (1988). Comparative anatomy of the cochlea and auditory nerve in mammals. Hear. Res..

[12] Stypulkowski P.H., van den Honert C. (1984). Physiological properties of the electrically stimulated auditory nerve. I. Compound action potential recordings. Hear. Res..

[13] Lai W.K., Dillier N. (2000). A simple two-component model of the electrically evoked compound action potential in the human cochlea. Audiol. Neuro-Otol..

[14] Dong Y., Briaire J.J., Biesheuvel J.D., Stronks H.C., Frijns J.H.M. (2020). Unravelling the temporal properties of human eCAPs through an iterative deconvolution model. Hear. Res..

[15] Esfandiari R.S., Bei L. (2018). Modeling and Analysis of Dynamic Systems.

[16] van den Honert C., Stypulkowski P.H. (1984). Physiological properties of the electrically stimulated auditory nerve. II. Single fiber recordings. Hear. Res..

[17] Rask-Andersen H., Liu W. (2015). Auditory nerve preservation and regeneration in man: relevance for cochlear implantation. Neural Regen. Res..

